# The Epidemiology, Phylogeny, and Strain Antigenicity of an Influenza A/H3N2 Virus Outbreak Among Vaccinated US Navy Midshipmen

**DOI:** 10.1111/irv.70184

**Published:** 2025-11-28

**Authors:** Simon D. Pollett, Emily Hone, Stephanie A. Richard, Kat Schmidt, Mark P. Simons, Michele Wayman, Jennifer Rothenberg, Vivian Hogan, Robert J. O'Connell, Timothy H. Burgess, Daniel Ewing, Appavu K. Sundaram, Anthony C. Fries, Drake H. Tilley, Rhonda E. Colombo

**Affiliations:** ^1^ Infectious Disease Clinical Research Program, Department of Preventive Medicine and Biostatistics Uniformed Services University of the Health Sciences Bethesda Maryland USA; ^2^ The Henry M. Jackson Foundation for the Advancement of Military Medicine, Inc Bethesda Maryland USA; ^3^ Agile Vaccines and Therapeutics Department Naval Medical Research Command Silver Spring Maryland USA; ^4^ United States Air Force School of Aerospace Medicine (USAFSAM), Wright‐Patterson Air Force Base Dayton Ohio USA; ^5^ Naval Health Clinic Annapolis Annapolis Maryland USA; ^6^ Department of Medicine Uniformed Services University of the Health Sciences Bethesda Maryland USA

**Keywords:** epidemiology, influenza A/H3N2, outbreak, phylogeny, US navy, vaccinated

## Abstract

We performed epidemiological, genetic, and antigenic characterization of an influenza A/H3N2 outbreak in a congregate setting of young adults with high vaccination rates. We noted substantial duty‐days lost, rapid spread, and vaccine antigenic divergence. The risk of influenza in healthy vaccinated adults underscores the need to develop universal influenza vaccines.

## 1 Introduction

Seasonal influenza has re‐emerged after the COVID‐19 pandemic to cause a substantial annual burden of infections, morbidity, and mortality [1]. While influenza vaccination is effective against severe illness, particularly in those who are older or with comorbidities, it remains only moderately effective at preventing infection and transmission [1]. Influenza vaccine effectiveness (VE) against infection typically ranges between 40% and 60%, but this varies by age, comorbidities, vaccine strain match, and timing of vaccination [1]. Meta‐analyses have shown that VE is typically lower for A/H3N2 compared to other subtypes [2].

Limited VE against infection and transmission may reduce the benefits of influenza vaccination for younger, lower‐risk adults in whom vaccination is either recommended or required, including healthcare workers and the active duty component of the US military [3, 4]. Insights into the functional burden (e.g., days of work lost) and drivers of influenza spread (e.g., vaccine immune escape) in such highly vaccinated and typically younger groups may support the optimization of influenza prevention strategies, including the development of universal influenza vaccines that prevent infection and sequelae across all ages and strains [5].

## 2 Methods

To this end, we leveraged an acute respiratory infection (ARI) surveillance study at the United States Naval Academy (USNA) to characterize the epidemiology, clinical and functional outcomes, phylogeny, and strain antigenicity of a recent influenza A/H3N2 outbreak among vaccinated midshipmen (undergraduate students). “Acute Respiratory Infections at the Academy” (ARIA) is a study of medically attended ARIs (MAARIs) among USNA midshipmen and staff presenting to the local brigade medical unit (BMU) for clinical care. The USNA (Annapolis, MD) comprises ≈4400 midshipmen across 4 years of undergraduate school who receive all of their primary care at the BMU. Midshipmen reside on campus in a congregate living‐training environment and are all required to receive annual influenza vaccination, unless medically or administratively exempt [4].

ARIA abstracts clinical and microbiological data from MAARI patients who present for care at the BMU (see Supporting Information and [6]). These data are abstracted from electronic medical records and include clinical diagnoses, on‐site respiratory microbiological test results, symptom type and duration, sick‐in‐quarters (SIQ) assignment, hospitalization, treatment, and vaccination history. In addition, the residential location of MAARI patients and the time‐varying infection control posture of the USNA (e.g., masking and case isolation) are recorded. Residual clinical respiratory tract swabs (including residual SARS‐CoV‐2 rapid tests) undergo multiplex PCR and sequencing of respiratory viruses, including influenza viruses (see Supporting Information). Respiratory swabs that are PCR positive for influenza with a cycle threshold value ≤ 36 undergo whole genome sequencing, with determination of CDC clade and antiviral resistance markers.

In response to this A/H3N2 outbreak, we performed extended epidemiological, virological, and antigenic analyses of influenza cases (see Supporting Information). This included the estimation of influenza A/H3N2 *R*_*t*_ before and after outbreak control measures. Phylogenies of outbreak A/H3N2 sequence strains and representative background sequences (including 2023–2024 Northern Hemisphere influenza A/H3N2 vaccine strains) were inferred [7]. We performed phylogenetic trait clustering analysis to identify if A/H3N2 cases clustered within either a specific graduation year and/or residence hall wing location (see Supporting Information). Other sequence analyses included amino‐acid comparison of infecting A/H3N2 sequences against 2023–2024 A/H3N2 vaccine strain HA1 sequences.

Finally, we performed antigenicity analysis on influenza A/H3N2 viral isolates cultured from a subset of PCR‐positive respiratory swabs. Ferret antisera were generated to A/H3N2 influenza strains included in the FDA‐approved 2023–2024 Northern Hemisphere egg (A/Darwin/9/2021‐like) and cell/recombinant‐based (A/Darwin/6/2021‐like) vaccines [8]. Ferret antisera neutralization titers to outbreak viral isolates were performed using CDC's high‐content imaging‐based neutralization test assay [9]. The proportion of influenza outbreak strains with a drop in neutralization titers compared to reference vaccine strains was determined.

The study protocol was approved by the USU Institutional Review Board in compliance with all applicable Federal research regulations governing the protection of human subjects as prescribed in 45 CFR 46.

## 3 Results

**FIGURE 1:**
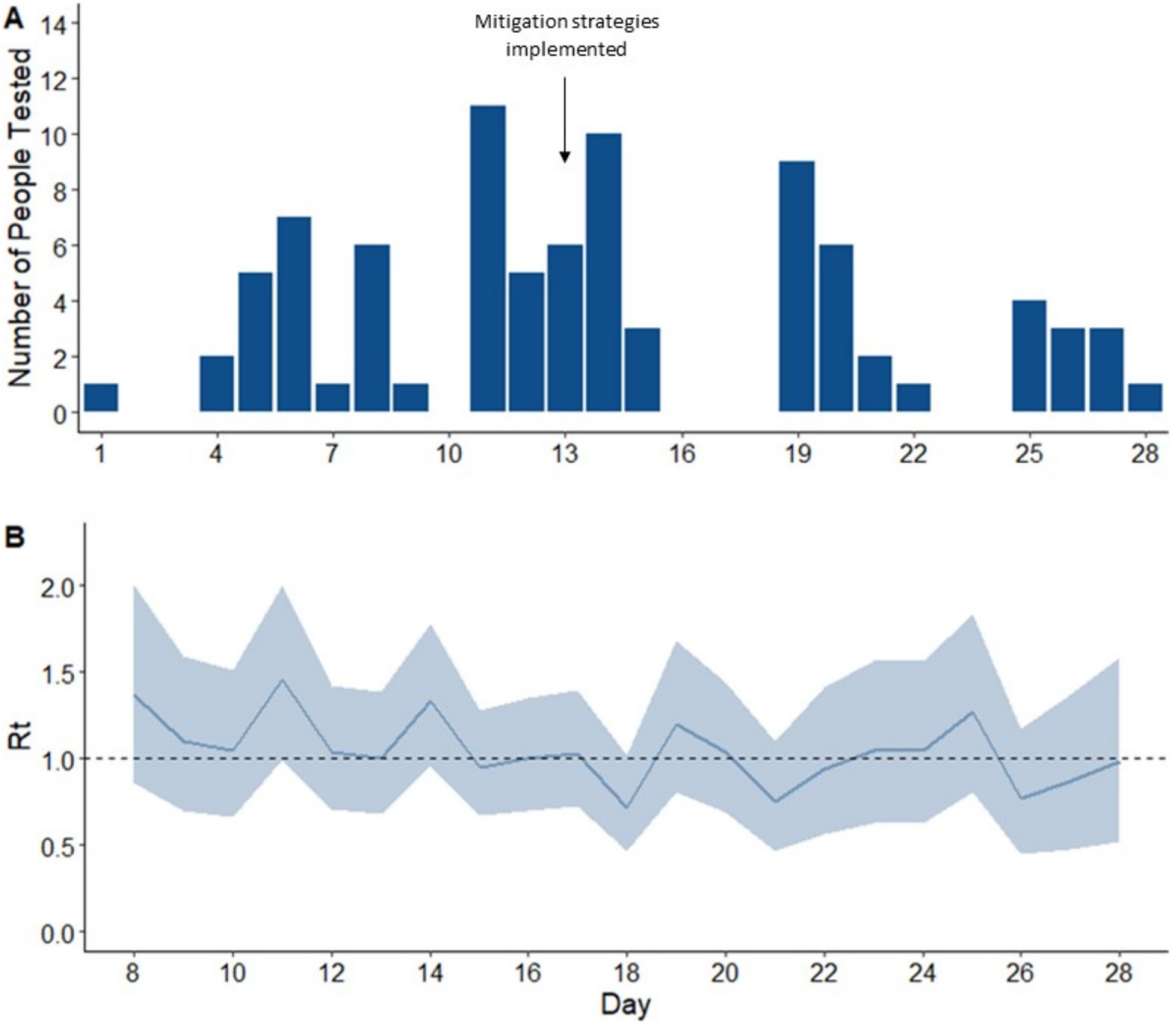
(A) Influenza A/H3N2 case counts among USNA Midshipmen and Staff Evaluated in February 2024. Day 1 refers to the collection time of the first influenza A/H3N2 outbreak clonal sequence. “Mitigation strategies implemented” included early syndromic treatment, case isolation, case masking, and contact chemoprophylaxis. (B) Estimated *R_t_* (effective reproductive number) of influenza A/H3N2 virus cases in February 2024. Day 1 refers to the collection timing of the first clonal influenza sequence.

**FIGURE 2:**
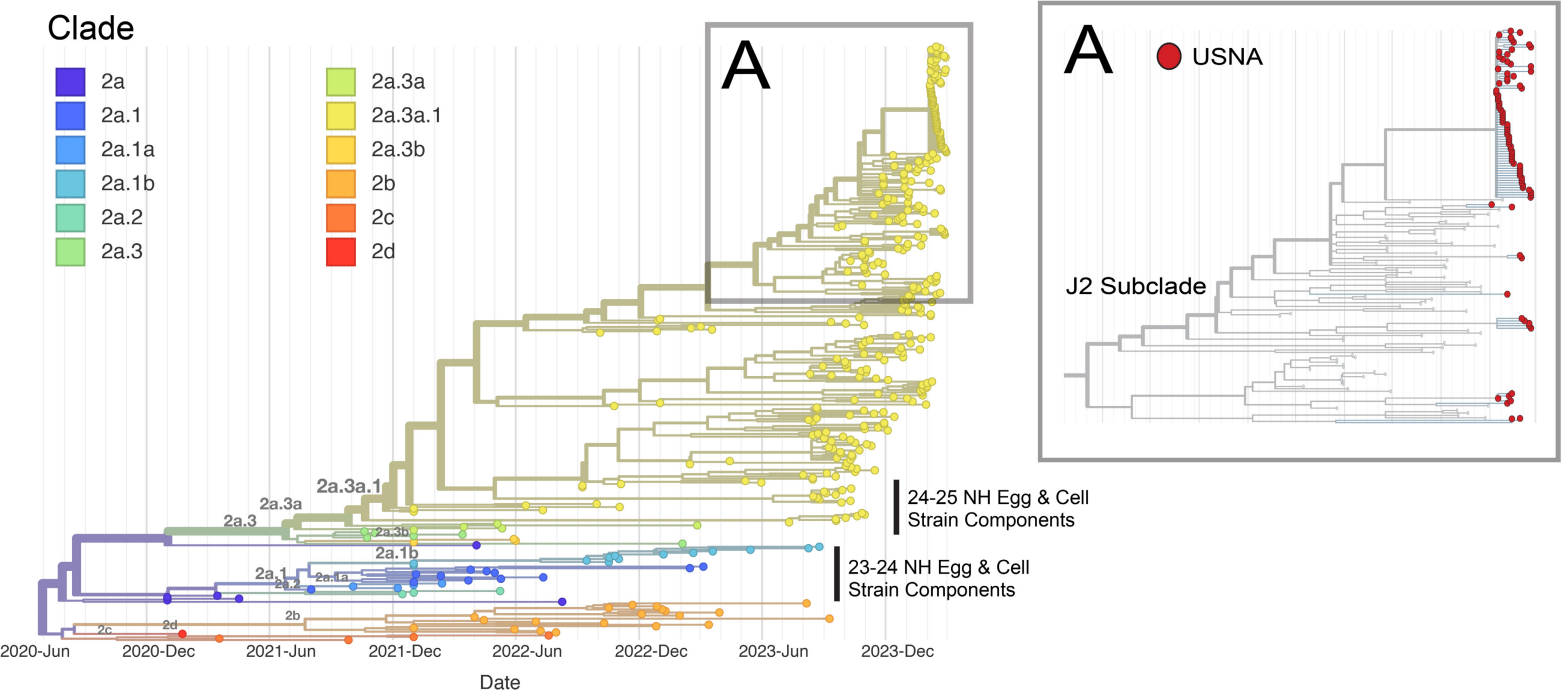
Time‐scaled phylogeny of influenza A/H3N2 HA segment genes. Clade numbers are annotated by color following the inset legend. Inset A captures the diversity of 2a.3a.1 strains from influenza infections in this February 2024 US Naval Academy (USNA) outbreak investigation in addition to non‐outbreak USNA H3N2 infection sequences from January and March 2024 (red tips), with gray tips for background sequences from GISAID. The 2023–2024 and 2024–2025 Northern Hemisphere (NH) vaccine strains sequences are indicated.

An influenza A/H3N2 outbreak emerged in February 2024 at the USNA, with declining case trends and *R* *_t_* following mitigation strategies (case masking, isolation, and post‐exposure oseltamivir; Figure 1). A total of 84 A/H3N2 cases were recorded, predominantly in midshipmen (Table 1), reflecting a medically attended influenza attack rate of 1.95% (95% CI: 1.56%–2.41%). HA sequence data from these cases confirmed local clonal transmission (3C.2a1b.2a.2a.3a.1 clade), consistent with early *R*_*t*_ estimates (Figures 1 and 2). The median age of cases was 20 years, and the majority were male and white.

**TABLE 1:** Demographic, clinical, and viral characteristics of Influenza A/H3N2 positive cases.

	All (*N* = 84)^a^
Age (years)	
Median [IQR]	20 [19, 21]
Range	18–25
Sex: male	53 (63%)
Race (*N* = 75)	
White	53 (71%)
Black	11 (15%)
Asian	8 (11%)
Islander/Indian/Alaska Native	3 (4%)
Ethnicity: not Hispanic or Latino	77 (92%)
Midshipmen	83 (99%)
Number of SIQ/isolation days assigned (*N* = 78)	
Median [IQR]	3 [1, 4]
Range	1–5
A/H3N2 clade	
3C.2a1b.2a.2a.3a.1	84 (100%)
Viral antiviral resistance mutation^b^	
None detected	80 (95%)
PA none detected, NA sequence fail	2 (2%)
NA fail and PA sequence fail	2 (2%)
Oseltamivir prescription during episode	47 (56%)
Vaccinated 9/2023–2/2024	77 (92%)^c^
IIV: Egg‐based	76 (91%)
Recombinant (Flublok)	1 (1%)
Days from 2023 to 2024 influenza vaccine to initial influenza illness encounter	
Median [IQR]	125 [122, 132]
Range	112–165
Vaccinated for 2022–2023 influenza season	64 (91%)^d^

^a^ *N* (%) for categorical; median [IQR] and range for continuous; PA = polymerase segment sequence; NA = neuraminidase segment sequence.

^b^ Oseltamivir resistance inferred by R292K, E119V, and E119D; baloxavir resistance inferred by I38T.

^c^ Unvaccinated status may reflect missing electronic medical record vaccine status entry.

^d^ Able to assess in only 70 midshipmen, the remaining 14 were 1st‐year midshipmen, and earlier medical records were not available.

Phylogenetic trait analysis did not show evidence of clustering of A/H3N2 infections by either midshipmen year or residence wing (see Supporting Information). Specifically, the Parsimony Score (PS) and Association Index (AI) metric values from a Bayesian analysis of tip significance (BaTS) analysis indicated that tree tips do not exclusively cluster by residence wing and supported a scenario of viral spread between residence wings, as well as some local transmission within residence wings.

Of the recorded A/H3N2 cases, at least 77 (92%) received the 2023/2024 Northern Hemisphere influenza vaccine by November 2023; 76 received the egg‐based inactivated influenza vaccine, containing the A/Darwin/9/2021 (H3N2)‐like virus, and one received the recombinant vaccine containing the A/Darwin/6/2021 (H3N2)‐like virus [8]. Over half (56%) of cases were treated with oseltamivir, and no markers of antiviral resistance were detected. No cases were hospitalized, and the median sick‐in‐quarters time was 3 days (Table 1).

Phylogenetic analysis indicated that the outbreak 3C.2a1b.2a.2a.3a.1 clade differed from the clade of the 2023–2024 Northern Hemisphere vaccine A/H3N2 HA‐egg‐based vaccine sequence (A/H3N2 Darwin 9) (Figure 2), including at immunologically relevant amino acid residues (see Supporting Information). These amino acid residues were located in regions of known antigenicity and neutralization functional significance (see Supporting Information), consistent with World Health Organization 3C.2a1b.2a.2a.3a.1 clade phenotyping, which noted that these strains (which typically contained the E50K, G53N, N96S, I140K, I192F, and I223V residues noted in the Supporting Information) had a significant reduction of neutralization by Darwin 9 2023–2024 egg vaccine strain antisera [10]. Ferret antigenicity analysis on the strains isolated from this USNA outbreak showed that 85% of the infecting A/H3N2 outbreak strains had a fourfold or lower HA titer relative to the 2023–2024 egg‐based vaccine strain (Table 2).

**TABLE 2:** Percent of 34 Influenza A/H3N2 strains (isolated from 34 US Navy Personnel) with HA titer fold changes relative to 2023–2024 Northern Hemisphere A/H3N2 vaccine reference strains.

Titer fold drop of infecting strain from reference vaccine strain	*N* (%) with titer drop from Darwin 6 2023–2024 cell/recombinant Northern Hemisphere H3N2 reference vaccine strain	*N* (%) with titer drop from Darwin 9 2023–2024 egg Northern Hemisphere H3N2 reference vaccine strain
1:2	1 (3%)	4 (12%)
1:4	0	28 (82%)
1:8	0	0
1:16	0	1 (3%)

## 4 Conclusions

Our characterization of the burden of this outbreak emphasizes that influenza A/H3N2 infections can be impactful, with substantial attack rates (≈1 in 50 midshipmen presenting for medical care) and work days lost (median 3 days) in fully vaccinated and generally healthy congregate populations. Nonpharmaceutical countermeasures and chemoprophylaxis appeared effective in mitigating this outbreak, and antiviral resistance was not seen. This outbreak timing corresponded with the largest number of A/H3N2 cases reported elsewhere in the United States during the 2023–2024 influenza season (2024 EpiWeek 6–8), coincided with a peak of laboratory‐confirmed influenza cases in the State of Maryland [11, 12], and occurred after at least two other A/H3N2 outbreaks in other military congregate settings in 2023–2024 [13, 14].

Despite vaccination, military congregate settings are at risk for such outbreaks due to the close proximity of personnel, often combined with the stress of training or operations. In this outbreak, we showed evidence of substantial spatial viral mixing across the residential dormitory wings and among each class year group, illustrating how influenza viruses can rapidly spread across a congregate population. While we did not collect detailed data on activities, which may have linked specific cases (e.g., sports), these may have enabled influenza spread. The lack of available contact tracing data, sport practice, or dormitory‐level movement patterns is a limitation of our analysis, and incorporating such data could have explored potential drivers of influenza spread (beyond VE considerations).

The outbreak occurred predominantly in vaccinated midshipmen. While we ascertained too few unvaccinated to estimate VE in this study population, the frequency of vaccinated infections was consistent with CDC estimates of a moderate 2023–2024 Northern Hemisphere influenza vaccine VE against A/H3N2 outpatient infections (VE = 54% [95% CI: 11%–77%]) [15, 16, 17], and low VE (28% [95% CI: −1%–49%]) against A/H3N2 infections in US servicemembers derived from Armed Forces Health Surveillance Division 2023–2024 mid‐season VE estimates [18].

Our strain antigenicity analysis (using ferret antisera) suggested possible immune escape from the egg‐based 2023–2024 Northern Hemisphere vaccine component may have contributed to this outbreak. Vaccine‐elicited antibody waning may also have played a role (median time‐since‐vaccine of cases = 125 days), although we did not have human sera to test this hypothesis and ferret antisera panels could not be used to infer that immunity to H3N2 strains waned faster than H1N1 strains, particularly with undefined assay upper limits of neutralization.

Taken together, our findings underscore the risk and impact of influenza, even in the young and vaccinated, and highlight the challenges of selecting vaccine strains which offer protection across an entire influenza season. This outbreak characterization emphasizes the importance of ongoing efforts to develop newer vaccines that provide more universal coverage and improved efficacy against infection and transmission, as well as severe influenza disease [5]. These efforts could be supported by future cohort studies, which seek to elucidate correlates of vaccine protection, in addition to ongoing characterization of influenza infections and influenza vaccine VE in US military servicemembers.

## Author Contributions

**Simon D. Pollett:** conceptualization, supervision, writing – review and editing, writing – original draft, funding acquisition, resources, investigation, methodology. **Emily Hone:** formal analysis, data curation, visualization, writing – review and editing. **Stephanie A. Richard:** data curation, writing – review and editing, methodology, supervision. **Kat Schmidt:** writing – review and editing, conceptualization. **Mark P. Simons:** writing – review and editing. **Michele Wayman:** writing – review and editing, project administration. **Jennifer Rothenberg:** project administration, writing – review and editing. **Vivian Hogan:** writing – review and editing, data curation. **Robert J. O'Connell:** writing – review and editing. **Timothy H. Burgess:** writing – review and editing. **Daniel Ewing:** writing – review and editing. **Appavu K. Sundaram:** writing – review and editing. **Anthony C. Fries:** writing – review and editing, data curation. **Drake H. Tilley:** writing – review and editing, project administration, supervision, investigation, methodology, resources. **Rhonda E. Colombo:** supervision, project administration, writing – review and editing, resources, funding acquisition, investigation, methodology, conceptualization.

## Disclosure

The contents of this publication are the sole responsibility of the author(s) and do not necessarily reflect the views, opinions, or policies of the Uniformed Services University of the Health Sciences (USU), the Department of Defense (DoD), the Departments of the Army, Navy, or Air Force, or the Henry M. Jackson Foundation for the Advancement of Military Medicine Inc. (HJF). Mention of trade names, commercial products, or organizations does not imply endorsement by the U.S. Government. Investigators followed human subjects protection 45 CFR 46 policies.

T.H.B., R.J.O., M.P.S., D.H.T., C.D.W, D.E., A.K.S., and A.C.F. are US Government employees or service members, and the work described was created as part of their official duties. Title 17 U.S.C. §101 defines a US Government body of work as a work created by an employee of the US Government or military service member as part of that person's official duties. Title 17 U.S.C. §105 reports “Copyright protection under this title is not available for any work of the United States Government.”

## Ethics Statement

The study protocol was approved by the USUHS Institutional Review Board in compliance with all applicable Federal research regulations governing the protection of human subjects as prescribed in 45 CFR 46.

## Conflicts of Interest

The authors declare no conflicts of interest.

## Supporting information


**Table S1:** Influenza vaccine strain inoculated ferret antisera neutralization titers to influenza A/H3N2 strains isolated from 34 US Navy Personnel.
**Table S2:** Amino acid substitutions in infecting viral HA1 sequences from 2024 USNA influenza infections relative to the Darwin 9 2023–2024 egg Northern Hemisphere H3N2 reference vaccine strain.
**Table S3:** Comparative influenza vaccine strain inoculated ferret antisera neutralization titers to influenza A/H3N2 strains isolated from 34 US Navy Personnel.

## Data Availability

Data for this study are available from the Infectious Disease Clinical Research Program (IDCRP), headquartered at the Uniformed Services University of the Health Sciences (USU), Department of Preventive Medicine and Biostatistics. Review by the USU Institutional Review Board is required for use of the data collected under this protocol. Furthermore, the dataset includes Military Health System data collected under a Data Use Agreement that requires accounting for uses of the data. Data requests may be sent to: Address: 6270B Rockledge Drive, Suite 340, Bethesda, MD 20817. Email: contactus@idcrp.org.

## References

[1] Centers for Disease Control and Prevention , “Benefits of the Flu Vaccine,” (2024) [cited 2024 November 22]; https://www.cdc.gov/flu‐vaccines‐work/benefits/?CDC_AAref_Val=https://www.cdc.gov/flu/vaccines‐work/vaccineeffect.htm.

[2] G. N. Okoli , F. Racovitan , T. Abdulwahid , C. H. Righolt , and S. M. Mahmud , “Variable Seasonal Influenza Vaccine Effectiveness Across Geographical Regions, Age Groups and Levels of Vaccine Antigenic Similarity With Circulating Virus Strains: A Systematic Review and Meta‐Analysis of the Evidence From Test‐Negative Design Studies After the 2009/10 Influenza Pandemic,” Vaccine 39, no. 8 (2021): 1225–1240. 33494964 10.1016/j.vaccine.2021.01.032

[3] Centers for Disease Control and Prevention , “State Immunization Laws for Healthcare Workers and Patients,” (2014) [cited 2024 November 22]; https://www2a.cdc.gov/vaccines/statevaccsApp/AdministrationbyVaccine.asp?Vaccinetmp=Influenza.

[4] Defense Health Agency , “Procedural Instruction Number 6025.34. Guidance for the DoD Influenza Vaccination Program (IVP),” (2020) [cited 2024 November 22], https://www.health.mil/Reference‐Center/DHA‐Publications/2020/08/21/DHA‐PI‐6025‐34.

[5] E. J. Erbelding , D. Post , E. Stemmy , et al., “A Universal Influenza Vaccine: The Strategic Plan for the National Institute of Allergy and Infectious Diseases,” Journal of Infectious Diseases 218, no. 3 (2018): 347–354. 29506129 10.1093/infdis/jiy103 PMC6279170

[6] K. Schmidt , S. D. Pollett , S. A. Richard , et al., “Opportunities for Enhanced Public Health Surveillance via Molecular Detection and Sequencing of Diverse Respiratory Viruses From Self‐Collected SARS‐CoV‐2 Antigen Test Swabs,” Open Forum Infectious Diseases 11, no. 8 (2024): ofae447. 39175525 10.1093/ofid/ofae447 PMC11339864

[7] J. Hadfield , C. Megill , S. M. Bell , et al., “Nextstrain: Real‐Time Tracking of Pathogen Evolution,” Bioinformatics 34, no. 23 (2018): 4121–4123. 29790939 10.1093/bioinformatics/bty407 PMC6247931

[8] U.S. Food and Drug Administration , “Influenza Vaccine for the 2023–2024 Season,” (2023) 2023 [cited 2024 November 22], https://www.fda.gov/vaccines‐blood‐biologics/lot‐release/influenza‐vaccine‐2023‐2024‐season.

[9] P. A. Jorquera , V. P. Mishin , A. Chesnokov , et al., “Insights Into the Antigenic Advancement of Influenza A(H3N2) Viruses, 2011‐2018,” Scientific Reports 9, no. 1 (2019): 2676. 30804469 10.1038/s41598-019-39276-1 PMC6389938

[10] World Health Organization , “Recommended Composition of Influenza Virus Vaccines for Use in the 2024 Southern Hemisphere Influenza Season,” (2023) [cited 2025 August 15], https://www.who.int/publications/m/item/recommended‐composition‐of‐influenza‐virus‐vaccines‐for‐use‐in‐the‐2024‐southern‐hemisphere‐influenza‐season.

[11] Centers for Disease Control and Prevention , “FluView,” (2024) [cited 2024 November 22], https://www.cdc.gov/fluview/?CDC_AAref_Val=https://www.cdc.gov/flu/weekly/index.htm.

[12] Maryland Department of Health , “MDH FluWatch,” (2025) [cited 2025 August 15] https://health.maryland.gov/phpa/influenza/pages/flu‐dashboard.aspx.

[13] Global Center for Health Security , “Influenza A Outbreak Puts Bogotá Military Academy Under Strict Quarantine,” (2024) [cited 2024 November 22], https://www.unmc.edu/healthsecurity/transmission/2024/02/06/influenza‐a‐outbreak‐puts‐bogota‐military‐academy‐under‐strict‐quarantine/.

[14] J. M. Velasco , M. T. Valderama , P. C. Diones , et al., “Outbreak of Influenza and SARS‐CoV‐2 at the Armed Forces of the Philippines Health Service Education and Training Center, September 25‐October 10, 2023,” MSMR 31, no. 5 (2024): 9–15. PMC11189823 38847656

[15] S. Zhu , J. Quint , T. M. León , et al., “Interim Influenza Vaccine Effectiveness Against Laboratory‐Confirmed Influenza—California, October 2023‐January 2024,” MMWR. Morbidity and Mortality Weekly Report 73, no. 8 (2024): 175–179. 38421946 10.15585/mmwr.mm7308a4 PMC10907038

[16] A. M. Frutos , A. M. Price , E. Harker , et al., “Interim Estimates of 2023‐24 Seasonal Influenza Vaccine Effectiveness—United States,” MMWR. Morbidity and Mortality Weekly Report 73, no. 8 (2024): 168–174. 38421935 10.15585/mmwr.mm7308a3 PMC10907036

[17] Centers for Disease Control and Prevention , “CDC Seasonal Flu Vaccine Effectiveness Studies,” (2024) [cited 2024 November 22], https://www.cdc.gov/flu‐vaccines‐work/php/effectiveness‐studies/.

[18] A. C. Fries , “DoD Influenza Surveillance and Mid‐Season Vaccine Effectiveness,” (2024) [cited 2025 August 15] https://www.fda.gov/media/176781/download.

