# Supplementary material for: The Epidemiology, Phylogeny, and Strain Antigenicity of an Influenza A/H3N2 Virus Outbreak Among Vaccinated US Navy Midshipmen
Source: Influenza Other Respir Viruses. 2025 Nov 28;19(12):e70184. doi: 10.1111/irv.70184 (PMC12981523; doi:10.1111/irv.70184)
Supplement: Supplementary file 1 — Table S1: Influenza vaccine strain inoculated ferret antisera neutralization titers to influenza A/H3N2 strains isolated from 34 US Navy Personnel. Table S2: Amino acid substitutions in infecting viral HA1 sequences from 2024 USNA influenza infections relative to the Darwin 9 2023–2024 egg Northern Hemisphere H3N2 reference vaccine strain. Table S3: Comparative influenza vaccine strain inoculated ferret antisera neutralization titers to influenza A/H3N2 strains isolated from 34 US Navy Personnel. [file IRV-19-e70184-s001.docx]

**Supplementary Materials:** The epidemiology, phylogeny, and strain antigenicity of an influenza A/H3N2 virus outbreak among vaccinated U.S. Navy midshipmen

**Supplementary methods:**

*Epidemiological analysis methods:*

R*_t_* estimation was based on influenza case initial encounter date. We utilized the *EpiEstim* R package (v2.2-4) and prior parameter estimates mean R_t_ 1.47 (95% CI = 1.30 - 1.72) gathered from Archer *et.al* 2012 (*1, 2*). Attack rate 95% confidence intervals were estimated using the binomial exact method. All epidemiological analyses were performed in R Studio (Version 2023.03.0) or using Stata v14.0 (StataCorp, College Station, TX).

*Molecular testing of residual clinical specimens:*

Provider collected nasopharyngeal (NP) swabs were stored at -80°C and shipped to the United States Air Force School of Aerospace Medicine (USAFSAM) on a weekly basis in viral transport media. At USAFSAM, all (NP) swabs underwent influenza testing via the Luminex NxTAG Respiratory Pathogen Panel (Diasorin, Italy) as well as the Centers for Disease Control and Prevention (CDC) Influenza SARS-CoV-2 (FluSC2) Multiplex Assay (CDC, https://archive.cdc.gov/www_cdc_gov/coronavirus/2019–ncov/lab/multiplex.html).

*Influenza virus sequencing:*

Influenza whole genome virus sequencing was performed on clinical residual nasopharyngeal PCR swabs with a CDC FluSC2 cycle threshold (Ct) value ≤36 for influenza A or influenza B using the Illumina NextSeq 2000 platform. As described by Schmidt et al., we used a universal primer sequencing approach for influenza A and for influenza B viruses (*3-5*) with further amplicon sequence assembly using the Iterative Refinement Meta Assembler (IRMA) (*6, 7*). The accession numbers for all study HA gene influenza sequences were deposited in GenBank with the following accession numbers PQ412847 - PQ412933.

*Influenza virus sequence analysis, including phylogenetic analysis:*

Influenza A/H3N2 study sequences underwent clade typing using Nextclade v3.8 (*8*). Additionally, we determined the proportion of study sequences with known antiviral resistance markers present. Phylogenetic analysis was performed using outbreak A/H3N2 HA segment sequence strains and representative background GISAID influenza A/H3N2 HA segment sequences from <https://nextstrain.org/seasonal-flu> (*8*) which included HA gene sequences from the 2023-2024 Northern Hemisphere egg and cell based vaccine A/H3N2 HA strain sequences (A/Darwin/9/2021-like and A/Darwin/6/2021-like sequences) (*8*).

HA gene sequence alignment and tree inference using IQTree were performed in the NextStrain environment using default settings (*9*). Resultant trees were visualized in Auspice (*9*). We measured the correlation of graduation year and residence hall to determine if the observed cases clustered within cohorts at the USNA. We used the Bayesian analysis of tip significance (BaTS v0.9.0) tool following Parker et al. 2008 to estimate the Parsimony Score (PS) and Association Index (AI) metrics (*10*). We measured these statistics using a sampling of 100 posterior tree topologies using an HKY substitution model in BEAST v1.10.4 capturing phylogenetic uncertainty in the USNA outbreak (*11*).

Additionally, we curated an alignment of outbreak influenza A/H3N2 HA1 and the 2023-2024 Northern Hemisphere egg based vaccine strain sequence (A/Darwin/9/2021) and performed sequence comparison restricted to immunophenotype annotated amino acids *(12, 13).* Sequence comparison was performed using Geneious 2024.0.7 software (GraphPad Software LLC, Boston, MA).

*Virus Propagation for HINT:*

The clinical specimens were isolated by seeding 6 x 10^5 MDCK-SIAT1 cells (Sigma Cat# 05071502) into a T25 vented flask with 5 ml of complete media (DMEM (Gibco Cat# 11965-092) + 10% fetal bovine serum (Hyclone Cat#SH30396.03) and 1% Pen-Strep (Quality Biological Cat# 120-095-721)) a day before. The flask was then washed twice with 5 ml of PBS. After that, 50 µl of human respiratory specimen was diluted into 450 µl of virus growth medium (VGM) (DMEM + 0.2% bovine serum albumin (BSA)(Gibco Cat# 15260-037) + 1% Pen-Strep + 25 mM HEPES (Quality Biological Cat# 118-089-721)+ 3 µg/ml (TPCK)-trypsin(Accurate Chemical Cat# YCC340122) and incubated for 1 hour at 35°C with 5% CO2. Following this, 5 ml of VGM was added, and the mixture was incubated for 24-48 hours until cytopathic effects (CPE) were visible. The media and cells were then collected, centrifuged at 2,000 RPM for 10 minutes to clarify, aliquoted, and stored at -80°C.

*Virus Titration for HINT:*

Viruses are diluted in VGM without TPCK-trypsin (VGM-T) in a black, clear bottom plate (Corning #3603) in 50 µl final volume, starting from a concentration of 10^1 to 10^8 per well. Then, 50 µl of VGM-T is added to all wells, and 100 µl is added to the negative control wells. The plate is then placed in an incubator at 37°C with 5% CO2 for 1 hour. After that, 50 µl of cells at a concentration of 5 x 10^5 cells/ml is added to each well, and the plate is returned to the incubator for 20-24 hours. After incubation, the cells are fixed by removing the media, adding 100 µl of cold fixation solution (95% Methanol/5% Acetic Acid), and incubating for 1 hour at -20°C. Following this, the fixation media is removed, and the plates are washed 3 times with 200 µl/well of 1x PBS. Then, 50 µl of anti-NP antibody (Millipore Cat# MAB8251) (diluted at 1:1000 in 1% BSA/1x PBS) is added to each well and incubated overnight at 4°C. The next day, the plates are washed with 1x PBS and 50 µl of secondary antibody (1:1000 dilution of goat anti-mouse AlexaFluor 555 (Invitrogen Cat# A21424) in 4µM Hoechst solution (AnaSpec, Inc. Cat# 33258) in 1% BSA/1x PBS) is added. The plates are then incubated at room temperature for 1.5 hours, the solution is discarded, and the plates are washed 3 times with 1x PBS. Subsequently, 100 µl of 1x PBS is added to each well, the plates are covered and then read on Nexcelom Celigo. The aim is to determine the correct virus dilution that will yield approximately 1,500 infected cells per well (ICP/well), within a validated range of 300-3000 ICP/well.

*HINT Assay:*

A panel of antiserum was created by infecting ferrets with different strains of influenza. The antiserum was diluted starting at a ratio of 1:40 and serially diluted using VGM-T in black, clear bottom plates with a final volume of 50 µl. Next, 50 µl of diluted virus (at the previously determined titer that yields 1,500 ICP/well) was added to all dilutions and control wells. The plate was then incubated at 37 °C with 5% CO2 for 1 hour. Following this, 50 µl of 5 x 10^5 cells/ml were added to all wells and the plate was incubated for 20-24 hours at 37 °C with 5% CO2. After incubation, the plates were fixed, stained, and analyzed as described above for Virus Titration for HINT. The 50% inhibition was determined by calculating the average of the virus control wells and dividing by two.

**Supplementary results:**

*Bayesian phylogenetic trait analysis:*

We observed AI scores of 8.48 (7.93-9.08, p=0.57) and 8.68 (7.75-9.17, p=0.950) for residency and graduation class, respectively. Lower AI scores (e.g. <1) indicate strong clustering support. We observed PS scores of 54.54 (54.0-55.0, p=0.77) and 53.72 (53.0-55.0, p=0.88) for residency and graduation class, respectively. Low PS scores (e.g. 1< PS < n) indicate strong clustering support. The distribution of cohort traits across the tips of the tree are consistent with the weak support for clustering in these analyses.

| **Table S1.** Influenza vaccine strain inoculated ferret anti-sera neutralization titers to influenza A/H3N2 strains isolated from 34 U.S Navy Personnel | | |
| --- | --- | --- |
|  | Darwin 6/H3N2 | Darwin 9/H3N2 |
| Reference strain titers | 640 | 20480 |
| Study viral isolate  (double coded) |  |  |
| 1 | 640 | 5120 |
| 2 | 640 | 5120 |
| 3 | 640 | 5120 |
| 4 | 640 | 5120 |
| 5 | 640 | 5120 |
| 6 | 640 | 5120 |
| 7 | 640 | 5120 |
| 8 | 640 | 5120 |
| 9 | 640 | 5120 |
| 10 | 640 | 5120 |
| 11 | 640 | 5120 |
| 12 | 640 | 5120 |
| 13 | 640 | 5120 |
| 14 | 640 | 10240 |
| 15 | 640 | 10240 |
| 16 | 640 | 5120 |
| 17 | 640 | 5120 |
| 18 | 640 | 5120 |
| 19 | 640 | 5120 |
| 20 | 640 | 5120 |
| 21 | 640 | 5120 |
| 22 | 640 | 5120 |
| 23 | 1280 | 5120 |
| 24 | 1280 | 5120 |
| 25 | 1280 | 5120 |
| 26 | 320 | 5120 |
| 27 | 640 | 5120 |
| 28 | 1280 | 5120 |
| 29 | 640 | 5120 |
| 30 | 640 | 5120 |
| 31 | 640 | 5120 |
| 32 | 640 | 10240 |
| 33 | 1280 | 10240 |
| 34 | 640 | 1280 |

| **Table S2.** Amino acid substitutions in infecting viral HA1 sequences from 2024 USNA influenza infections relative to the Darwin 9 2023-2024 egg Northern Hemisphere H3N2 reference vaccine strain. | | | | | |
| --- | --- | --- | --- | --- | --- |
| HA1 Amino Acid Position | Antigenic Site^a^ | Receptor Binding Site^b^ | Egg^c^ | Substitution | Frequency |
| 50 | C |  |  | E -> K | 1.00 |
| 53 | C |  |  | D -> N | 1.00 |
| 78 | E |  |  | G -> D | 0.02 |
| 96 | D |  |  | N -> S | 1.00 |
| 122 | A | Y |  | N -> D | 1.00 |
| 140 | A |  |  | I -> K | 1.00 |
| 144 | A |  |  | S -> N | 0.06 |
| 145 | A |  | Y | S -> N | 0.01 |
| 168 | A |  |  | M -> I | 0.06 |
| 186 | B |  | Y | N -> D | 1.00 |
| 192 | B |  |  | I -> F | 1.00 |
| 207 | D |  |  | K -> R | 0.02 |
| 223 |  | Y |  | I -> V | 1.00 |
| 225 |  | Y | Y | G -> D | 1.00 |
| 276 | C |  | Y | K -> E | 1.00 |

^a^Antigenic sites - regions involved in antibody binding first described for A/Hong Kong/1/68 in Wiley et al. 1981 *(12)*, in addition to H3N2 viral neutralization significance as described in Broecker et al. 2018 (14) ]*.* Site A – Protruding loop forming center of most obvious antibody binding site; Site B – Alpha helix along the upper edge of a pocket involved in receptor binding activity and proposed as primary site of immunodominance; Site C – Bulge in structure at disulfide bond; Site D – Site not directly exposed to as antibody binding site but structurally impacts remote or adjacent regions at the interface of binding; Site E: here defined as additional locations known to be involved in additional binding sites when adjacent locations have a specific amino acid composition. Receptor Binding Site – amino acid positions involved or adjacent to involvement in receptor binding. ^c^Egg - amino acid positions subject to substitutions when passaged through eggs.

| **Table S3. Comparative influenza vaccine strain inoculated ferret anti-sera neutralization titers to influenza A/H3N2 strains isolated from 34 U.S Navy Personnel** | | | | | | |
| --- | --- | --- | --- | --- | --- | --- |
| Reference strain | Reference strain circulation year, subclade, clade and strain name | | | | | |
|  | 2024-2025 | 2024-2025 | 2021-2022 | 2021-2022 | 2020-2021 | 2020-2021 |
|  | H | H | F.1 | F.1 | D | D |
|  | 3C.2a1b.2a.2a.3a.1 | 3C.2a1b.2a.2a.3a.1 | 3C.2a1b.2a.1a | 3C.2a1b.2a.1a | 3C.2a1b.1b | 3C.2a1b.1b |
|  | Thailand 8 | Mass 18 | Tasmania egg | Tasmania cell | HK 2671 | HK 45 |
| 1 | 1280 | 1280 | 640 | 640 | 320 | 640 |
| 2 | 1280 | 1280 | 640 | 320 | 160 | 320 |
| 3 | 2560 | 1280 | 1280 | 640 | 320 | 640 |
| 4 | 2560 | 1280 | 640 | 640 | 320 | 1280 |
| 5 | 2560 | 2560 | 1280 | 640 | 320 | 640 |
| 6 | 2560 | 2560 | 1280 | 320 | 640 | 1280 |
| 7 | 2560 | 1280 | 640 | 320 | 320 | 640 |
| 8 | 2560 | 1280 | 1280 | 640 | 640 | 1280 |
| 9 | 5120 | 2560 | 1280 | 1280 | 640 | 1280 |
| 10 | 2560 | 1280 | 1280 | 640 | 320 | 640 |
| 11 | 2560 | 1280 | 640 | 640 | 320 | 640 |
| 12 | 2560 | 1280 | 1280 | 320 | 320 | 640 |
| 13 | 2560 | 1280 | 1280 | 640 | 320 | 640 |
| 14 | 2560 | 2560 | 2560 | 1280 | 1280 | 2560 |
| 15 | 5120 | 2560 | 1280 | 1280 | 640 | 1280 |
| 16 | 5120 | 2560 | 1280 | 1280 | 640 | 1280 |
| 17 | 2560 | 2560 | 1280 | 640 | 640 | 640 |
| 18 | 2560 | 2560 | 1280 | 640 | 640 | 1280 |
| 19 | 2560 | 2560 | 2560 | 2560 | 1280 | 2560 |
| 20 | 5120 | 2560 | 1280 | 1280 | 1280 | 2560 |
| 21 | 2560 | 2560 | 1280 | 1280 | 640 | 640 |
| 22 | 2560 | 2560 | 1280 | 1280 | 640 | 1280 |
| 23 | 5120 | 5120 | 2560 | 2560 | 2560 | 2560 |
| 24 | 5120 | 2560 | 2560 | 2560 | 1280 | 2560 |
| 25 | 2560 | 1280 | 1280 | 320 | 320 | 640 |
| 26 | 1280 | 1280 | 640 | 320 | 320 | 320 |
| 27 | 2560 | 1280 | 1280 | 640 | 320 | 1280 |
| 28 | 5120 | 5120 | 2560 | 2560 | 2560 | 2560 |
| 29 | 5120 | 2560 | 1280 | 1280 | 640 | 640 |
| 30 | 5120 | 2560 | 2560 | 2560 | 2560 | 2560 |
| 31 | 1280 | 1280 | 640 | 320 | 160 | 320 |
| 32 | 5120 | 5120 | 1280 | 1280 | 1280 | 320 |
| 33 | 2560 | 1280 | 1280 | 640 | 640 | 640 |
| 34 | 2560 | 1280 | 2560 | 320 | 160 | 640 |

**References:**

1. Archer BN, Tempia S, White LF, Pagano M, Cohen C. Reproductive number and serial interval of the first wave of influenza A(H1N1)pdm09 virus in South Africa. PloS One. 2012;7(11):e49482.

2. Cori A, Cauchemez S, Ferguson NM, Fraser C, Dahlqwist E, Demarsh A, et al. EpiEstim: Estimate Time Varying Reproduction Numbers from Epidemic Curves. R package version 2.2-4. 2021 [cited 2024 November 22]; <https://CRAN.R-project.org/package=EpiEstim>

3. Schmidt K, Pollett SD, Richard SA, Hogan V, Hone E, Rothenberg J, et al. Opportunities for enhanced public health surveillance via molecular detection and sequencing of diverse respiratory viruses from self-collected SARS-CoV-2 antigen test swabs. Open Forum Infect Dis. 2024;11(8):ofae447.

4. Zhou B, Donnelly ME, Scholes DT, St George K, Hatta M, Kawaoka Y, et al. Single-reaction genomic amplification accelerates sequencing and vaccine production for classical and Swine origin human influenza a viruses. J Virol. 2009;83(19):10309-13.

5. Zhou B, Lin X, Wang W, Halpin RA, Bera J, Stockwell TB, et al. Universal influenza B virus genomic amplification facilitates sequencing, diagnostics, and reverse genetics. J Clin Microbiol. 2014;52(5):1330-7.

6. Shepard SS, Meno S, Bahl J, Wilson MM, Barnes J, Neuhaus E. Viral deep sequencing needs an adaptive approach: IRMA, the iterative refinement meta-assembler. BMC Genomics. 2016;17(1):708.

7. Shepard SS, Meno S, Bahl J, Wilson MM, Barnes J, Neuhaus E. Erratum to: Viral deep sequencing needs an adaptive approach: IRMA, the iterative refinement meta-assembler. BMC Genomics. 2016;17(1):801.

8. Aksamentov I, Roemer C, Hodcroft EB, Neher RA. Nextclade: clade assignment, mutation calling and quality control for viral genomes. Journal of Open Source Software. 2021;6(67):3773.

9. Hadfield J, Megill C, Bell SM, Huddleston J, Potter B, Callender C, et al. Nextstrain: real-time tracking of pathogen evolution. Bioinformatics. 2018;34(23):4121-3.

10. Parker J, Rambaut A, Pybus OG. Correlating viral phenotypes with phylogeny: accounting for phylogenetic uncertainty. Infect Genet Evol. 2008;8(3):239-46.

11. Drummond AJ, Rambaut A. BEAST: Bayesian evolutionary analysis by sampling trees. BMC Evol Biol. 2007;7:214.

12. Wiley DC, Wilson IA, Skehel JJ. Structural identification of the antibody-binding sites of Hong Kong influenza haemagglutinin and their involvement in antigenic variation. Nature. 1981;289(5796):373-8.

13. Koel BF, Burke DF, Bestebroer TM, van der Vliet S, Zondag GCM, Vervaet G, et al. Substitutions Near the Receptor Binding Site Determine Major Antigenic Change During Influenza Virus Evolution. Science. 2013;342(6161):976-9.

14. Broecker F, Liu STH, Sun W, Krammer F, Simon V, Palese P. Immunodominance of antigenic site B in the hemagglutinin of the current H3N2 influenza virus in humans and mice. J Virol. 2018;92(20):e01100-18.
